# A Review of Capillary Pressure Control Valves in Microfluidics

**DOI:** 10.3390/bios11100405

**Published:** 2021-10-19

**Authors:** Shaoxi Wang, Xiafeng Zhang, Cong Ma, Sheng Yan, David Inglis, Shilun Feng

**Affiliations:** 1 School of Microelectronics, Northwestern Polytechnical University, Xi’an 710072, China; shxwang@nwpu.edu.cn (S.W.); zhangxiafeng@mail.nwpu.edu.cn (X.Z.); 2 State Key Laboratory of Transducer Technology, Shanghai Institute of Microsystem and Information Technology, Chinese Academy of Sciences, Shanghai 200050, China; macong1@mail.sim.ac.cn; 3 School of Information Science and Technology, ShanghaiTech University, Shanghai 201210, China; 4 Institute for Advanced Study, Shenzhen University, Shenzhen 518060, China; shengyan@szu.edu.cn; 5 School of Engineering, Faculty of Science and Engineering, Macquarie University, Sydney, NSW 2109, Australia; david.inglis@mq.edu.au; 6 School of Electrical and Electronic Engineering, Nanyang Technological University, Singapore 639798, Singapore

**Keywords:** capillary pressure control valve (CPCV), microfluidics, passive valve

## Abstract

Microfluidics offer microenvironments for reagent delivery, handling, mixing, reaction, and detection, but often demand the affiliated equipment for liquid control for these functions. As a helpful tool, the capillary pressure control valve (CPCV) has become popular to avoid using affiliated equipment. Liquid can be handled in a controlled manner by using the bubble pressure effects. In this paper, we analyze and categorize the CPCVs via three determining parameters: surface tension, contact angle, and microchannel shape. Finally, a few application scenarios and impacts of CPCV are listed, which includes how CPVC simplify automation of microfluidic networks, work with other driving modes; make extensive use of microfluidics by open channel, and sampling and delivery with controlled manners. The authors hope this review will help the development and use of the CPCV in microfluidic fields in both research and industry.

## 1. Introduction

Microfluidic technology has made great progress in the past two decades, and is a significant feature in a wide range of scientific and industrial work [1,2]. Microfluidic processes have strong potential in biomedical applications due to their small volume of samples and reagents, high throughput, and potential for automation. After decades of development, microfluidic devices have moved partly from the laboratory to practical applications, such as cell separation [3,4], analytical reactions and detections [5,6], immunoassays [7,8,9], as well as polymerase chain reaction (PCR) [10,11,12].

As a part of controlling and regulating liquid flow in microfluidics, micro-valves are essential. They can also be reliable and inexpensive [13]. With the increasing complexity and scale of microfluidic systems, research into microvalve designs has grown and various types have been demonstrated [14]. The function of these valves can be divided into stop valves [15], check valves [16,17], delay valves [18], retention valves [14], trigger valves [19], and siphon valves [20]. In addition to the purpose of the valve, we can categorize valves by the mechanism of actuation, which can be either active or passive. Active valves require an external energy source such as electrostatic [21,22,23], electromagnetic [24,25], pneumatic [26], hydraulic or photothermal [27,28,29]. These energy sources can control fluid flow through the deformation of a boundary, as in electromechanical [30] and pneumatic valves [31]. The energy may also change the state of a boundary, for example, by melting ice [10], wax [32,33], or a hydrogel [34]. These valves are best suited for repeatedly administering the liquid, controlling the pressure of the liquid, or pumping the liquid, but their operation requires peripheral actuators that limit their use. In contrast, the passive valve does not require additional driving equipment, which is more conducive to the integration and miniaturization of equipment. Existing passive valves include capillary pressure control valves [35], capillary burst valves [36], siphon valves [20], retention valves [14], flap valves [37], and check valves [38,39,40].

In an active valve, flow stops until the barrier is removed by some external force. In a passive valve the forward flow is stopped by a change in Laplace pressure. The capillary pressure control valve (CPCV) is a kind of passive valve that relies on the Laplace pressure generated by the change of the liquid front meniscus concavity. It may also be referred to as the Laplace pressure control valve or capillary valve. CPCVs do not require external equipment and rely solely on Laplace pressure at the liquid interfaces, which depends on the surface tension, contact angle and channel shape. CPCVs have been widely used as flow control valves in various microfluidic systems, which further enhances the performance of micro devices and expands the functions available in an integrated microsystem [14,41].

Surface tension changes little for a given combination of fluid and gas at a constant temperature, so the CPCV can be divided based on their actuating mechanism: either contact angle changes or geometric changes. In the first group, the Laplace pressure is different in two adjacent locations because of hydrophilic and hydrophobic patterning [42,43,44,45]. In the second group, the Laplace pressure is different in two adjacent locations because of a change in geometry, typically an expansion, which changes the radius of curvature [15,46,47].

The stopping principle of these valves is that the liquid will be blocked due to a Laplace pressure change caused by a sudden change in the liquid front meniscus. To open it and restore flow, the existing meniscus needs to be rebuilt to remove additional pressure [36]. There are essentially two ways to do this. The first is to increase the fluid pressure beyond the bubble pressure, usually by increasing the pumping pressure. In a Lab on Disk system the pressure is increased by increasing the spin speed [48]. Through theoretical calculation and experimental analysis, a large number of articles have reported the relationship between break pressure and valve channel characteristics, including valve channel geometry shape and material characteristics [36,46,49,50,51]. The second way to open the valve is to bring fluid in from the stopped side of the valve. The merged meniscus can then pass the stop valve. This is known as a trigger valve [19,52].

A. Olanrewaju et al. [14] have recently reviewed capillary microfluidics and described the capillary networks. Among them, part of the contents are the collections of the CPCV examples including stop valve, capillary trigger valve, capillary soft valve, capillary retention valve, retention burst valve, and delay valve. Moreover, the physics theories governing capillary flow are described, and the concepts of capillary circuits are also introduced. In this review, we focus on CPCV descriptions through the whole article. We discussed the specific fundamental physics theories related to the CPCV in detail; categorized them based on the theory elements with new applications of each kind of valve; discussed the potential of real applications in industrial and academic fields. We analyzed the CPCV examples from three fundamental elements: surface tension, contact angle and different microchannel shape. We hope it can give a direction describing the specific phenomena theoretically and easily for other academic and industrial control valves research, which can have more useful applications.

There are four parts in this paper. Part 2 is for the categories of CPCV and different three determining parameters of Laplace pressure, including surface tension, contact angle as well as microchannel shape. Part 3 provides a review for different developments and applications of CPCV. Part 4 is for the conclusions and outlook.

## 2. Categories of CPCV

With the expansion of microfluidic technology, CPCV is more attractive as a kind of passive valve, the threshold pressure of the valve is affected only by the geometry and liquid properties of the device. As no moving parts are involved, these valves are easier to manufacture and less likely to clog than moving valves.

The bubble pressure (or Laplace pressure) is the difference in pressure between the inside and the outside of a curved surface, such as bubbles or droplets. The relationship between Laplace pressure (*P*) and surface tension is described by Young–Laplace equation as follows:

(1) $$\Delta P\equiv P_{inside}-P_{outside}=\gamma (\frac{1}{R_{1}}+\frac{1}{R_{2}})$$

The pressure is larger on the concave side of the meniscus (gas–liquid interface) than on the convex side. Where *γ* is the surface tension of the liquid. $R_{1}$ and $R_{2}$ are the principal radii of curvature of the meniscus related to the contact angle and microchannel shape, where $R=\frac{\gamma }{cos\theta_{c}}$. The CPCV is categorized from the following aspects: surface tension *γ*, contact angle *θ_c_*, and its channel shape.

### 2.1. Surface Tension (γ)

Surface tension is defined in a very pragmatic way: the tension between any two adjacent parts of a liquid surface that interact perpendicular to their unit length boundary is called surface tension (*γ*). Using an arbitrary line to divide the liquid level into two parts, surface tension can be understood as the pull of a molecule on one side on the other side per unit length. At each point on both sides of the line, there is a surface tension perpendicular to the line and tangent to the surface.

The surface tension of water/mineral oil can be calculated according to:

(2) γ[mN/m]=51.83−0.103T

where *T* is temperature in Celsius. If the room temperature *T* is 22 °C, surface tension is around 49.56 mN/m.

The surface tension can be caused by either the metallic bonds or the hydrogen bonds, while the former one has a larger effect. Surface tension may be altered locally through changing surface energy, such as addition of surfactants [53] or by applied sunlight or even lasers [54] or magnetic fields [55].

### 2.2. Contact Angle (θ)

**Figure 1:**
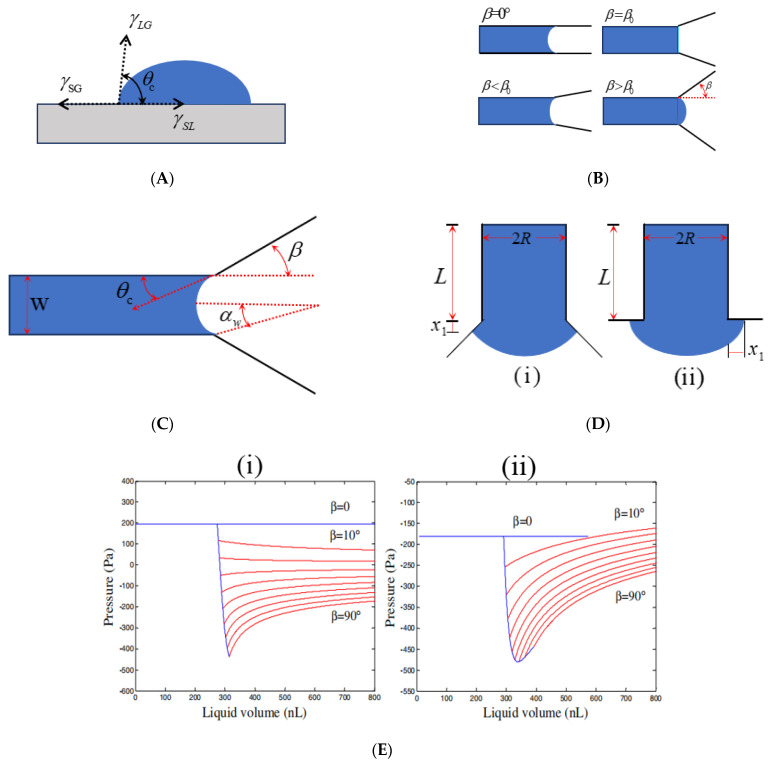
CPCV parameters and their relationships. (**A**) Definition of contact angle. (**B**) The relationship between the angle of triangle expansion β and the liquid level meniscus. (**C**) The common geometrical parameters in CPCV boundary pressure model are given. (**D**) Meniscus of axisymmetric channel in expansion regime bursting into divergent section, where advancement of liquid is described mainly by wetted length $x_{1}$ for (i) $\beta <90^{{}^{\circ}}$ and (ii) $\beta =90^{{}^{\circ}}$. (**E**) Variation in liquid pressure with liquid volume for hydrophilic and hydrophobic channels of R = 0.3 mm with expansion angles ranging from β = 0 to 90°, (**i**) hydrophilic channels interfacial properties γ=0.072 N/m and $\theta_{c}=66{}^{\circ}$; (**ii**) hydrophobic channels interfacial properties γ=0.072 N/m and $\theta_{c}=112{}^{\circ}$. Reprinted with permission from [56]. Copyright (2008) The Japan Society of Applied Physics.

In 1805, Thomas Young defined the contact angle at the solid–liquid–gas three-phase boundary. As shown in Figure 1A, the contact angle $\theta_{c}$ is the included angle from the solid–liquid interface through the liquid interior to the gas–liquid interface. The contact angle represents a method of showing liquid–solid adhesion.

The relation between interfacial tension and contact angle is given by Young’s equation:

(3) $$\gamma_{SG}=\gamma_{SL}+\gamma_{LG}cos\theta_{c}$$

where $\gamma_{SG,\;}\gamma_{SL}$ and $\gamma_{LG}$ are the interfacial tensions between solid and gas, solid and liquid, and liquid and gas, respectively.

#### 2.2.1. Hydrophobic/Hydrophilic

Since $\gamma_{SG}$ and $\gamma_{LG}$ are constant, it is known from the Young’s equation that the contact angle $\theta_{c}$ decreases accordingly when the surface tension between solid and liquid $\gamma_{SL}$ decreases, leading to the increase of adhesion energy standing for hydrophilic characteristics. Additionally, vice versa, the contact angle increases standing for the hydrophobic characteristics. A surface with droplet water contact angle greater than 90° is hydrophobic, but less than 90° is deemed hydrophilic. The affinity between a material and water is described by the term hydrophilic/hydrophobic. Materials with polar groups usually have great affinity for water and can attract water molecules to be easily wetted. On the contrary, hydrophobic materials tend to be non-polar with no affinity for water and are not easily wetted. For hydrophobic materials, when water comes into contact with the surface of the material, the contact angle is generally greater than 90°. Droplets that are large enough to experience gravitational forces that exceed the capillary forces are likely to break up into small droplets or beads. The opposite is true for hydrophilic materials, where small droplets will aggregate into a film. As the water molecule is polar, materials with polarizable surface groups tend to be hydrophilic, having contact angles that are less than 90°.

Common hydrophilic substances are aluminum, zinc, and other metals and their oxides, glass and mica, quartz, talc, calcite, quartz, and many other minerals. In addition, the single and associating -OH polar groups on the surface of the material, which can form hydrogen bonds with water molecules, are hydrophilic. The hydrophobic group is mainly represented by -NO_2_, Si-H, and Si-CHx groups, and the Si-F group also exists in a small amount [57], such as paraffin, Teflon (PTFE), polyamide (PA), PC (polycarbonate), PAN (polyacrylonitrile), fluorinated polyethylene, fluorocarbon wax, polyolefin, polyester, fluoro-free acrylates, and so on. Modifying the surface of the material to chang the hydrophilic and hydrophobic nature can change the contact angle, which is an important method to construct the surface tension valve.

Due to the capillarity property generated by surface tension, water is pulled into a microchannel with a hydrophilic surface inside, but it meets the stop barrier at the hydrophobic surfaces. Taking advantage of hydrophobic–hydrophilic interface (hydrophobic—more hydrophobic; hydrophilic—more hydrophilic) effect can be used to manage the flow of liquid in the microchannel [58].

#### 2.2.2. Material Properties

Material selection is the first step in the fabrication of microfluidic chips, which directly affects the function of chips and determines the processing and production methods of subsequent chips. Chips made of different materials have different production costs, processing difficulty and specific processing methods. The selection of chip materials is also related to the observation and detection of subsequent experiments. Different chip materials have a direct impact on the difficulty of optical detection in subsequent experiments due to their different light transmittance. CPCV is an important part of a chip, and different properties of different materials also directly affect the design and production of CPCV.

The materials for making microfluidic chips generally include silicon, glass and a variety of polymer materials, for example, polymethylmethacrylate (PMMA), polydimethylsiloxane (PDMS), polystyrene (PS), and Polycarbonate (PC), etc. Table 1 shows the advantages and weakness of common materials used to fabricate microfluidic chips, where the contact angles with water were summarized.

It can be seen from Table 1 that, compared with silicon and glass, low cost and easy processing are advantages of polymer materials, so polymer materials are mostly used in the production of microfluidic chips. PDMS is a widely used polymer material for microfluidic chip processing. PDMS is chemically inert and non-toxic; it has excellent light permeability and is convenient for optical detection; with good plasticity, it is easy to process; it is convenient for surface modification and bonding; it can be repeated many times for mass production. In addition, most of the polymer materials are naturally hydrophobic, such as PDMS (contact angle: 113.5 ± 2° [59]) and PMMA (contact angle: 97° [60]), and the surface performance is not stable. After hydrophilic treatment, there is the phenomenon of hydrophobic recovery, which will gradually lose hydrophilicity and return to hydrophobic state. This is very unfavorable for CPCV constructed by changing surface hydrophilicity and hydrophobicity, and seriously affects the application expansion of CPCV.

**Table 1:** Properties of different materials for preparing microfluidic chips.

Materials	Advantages	Weaknesses	Contact Angles with Water (°)
Silicon	Chemical inertness Smooth surface Mature technology Easy to mass production	Fragile High cost Opaque Complex surface chemistry	Silica (super-hydrophobic): 161 [61] Silica (conventional): 24.5 [61] Silicon (FDTS): 113.7 ± 3.1 [62] Silicon (DDMS):105 [63]
Glass	Good electroosmotic Good optical properties Easy for surface treatment	Fragile, high cost Bonding difficult Difficulty in a large aspect ratio	Glass (uncoated): 68.5 [64] Glass (2% APTES): 40 [65] Glass (MSNPs-CVD): 175 ± 2 [66] Glass (MSNPs-Sol): 158 ± 2 [66]
Polymer	Variety and low cost Transmission of light Easy to process and form Cheap mass production	Low heat-resistant Low thermal conductivity	PDMS: 113.5 ± 2 [59] PMMA: 97 [60] PDMS (APTES+MA): 60 [67]

#### 2.2.3. Surface Treatment Methods

The most commonly used materials for microfluidic devices, and therefore for surface tension valves, are glass and PDMS. The chemical stability of PDMS is very good, so its surface modification is quite difficult. In addition, the surface properties of PDMS are unstable. Even if the surface of PDMS becomes hydrophilic through modification, the surface will gradually lose hydrophilicity and return to the hydrophobic state, a phenomenon known as hydrophobic recovery [68,69]. The mechanism of PDMS surface instability is not yet clear. Most surface modification methods have not solved the problem of PDMS hydrophobic recovery. Common surface treatment methods include plasma treatment [70,71], covalent surfactants treatment [72], and Sol–gel coating [73,74].

**Plasma treatment:** this surface treatment method is currently the most commonly used method for surface modification of PDMS [69]. Plasma is an ionized gaseous substance composed of atoms deprived of some electrons and positive and negative ions generated after the ionization of atomic groups. It is often regarded as the fourth state of matter in addition to solid, liquid, and gas. In plasma treatment, oxygen plasma is used to react with the surface of the material to expose its hydrophilic chemical functional groups. When a surface is treated with hydrophilic plasma, it increases the surface energy of the object. When the surface energy of an object is high, its adhesion is high. However, plasma surface modification of PDMS can restore its hydrophobic properties within a few minutes, as exposed hydrophilic groups recombine with uncured hydrophobic polymer chains, causing the surface to lose hydrophilic properties. Maintaining the surface of the material in water immediately after plasma treatment or using solvent extraction to remove the uncured polymer can effectively slow down or prevent this recovery [70,71].

**Surfactants treatment:** surfactant molecules have charged “heads” and hydrophobic “tails” that can easily adsorb onto the hydrophobic surface and change its surface properties. Amphiphilic surfactant molecules enter the microchannel by running the buffer, the hydrophobic tail is physically adsorbed on the PDMS surface, and the hydrophilic surfactant head is extended into the buffer, making the PDMS surface hydrophilic in situ, to achieve the purpose of changing the surface properties of PDMS. This method not only reduces the cost of surface modification, but also makes it more rapid and simple. As the surfactant does not form a strong covalent bond with primary PDMS only by weak hydrogen bond binding, desorption can occur, and excessive surfactants are needed to dynamically supplement the desorption substances in the process, so as not to have a negative impact on the surface performance.

Nowadays, a large number of surfactants are applied to PDMS surface modification. For hydrophilic treatment, there is poly ethylene glycol methacrylate (PEGMA), 2-hydroxy ethyl methacrylate (HEMA), O2 plasma, tween-20 [75], 3-aminopropyl triethoxysilane (APTES) [67,76], polyvinyl alcohol (PVA) [72], polyvinylpyrrolidone (PVP) [77], Pluronic [78], etc. For hydrophobic treatment, there is octadecyltrichlorosilane (OTS) [79], Sigmacoat [80], Fluorosilane coupling agent, Saline, etc.

**Sol–gel coating:** in this method, the “solution” and “sol” of compounds containing high chemical active components are cured into a “gel” state through a series of treatments. Sol–gel technology can rapidly and repeatedly construct a large number of ordered hydrophilic and hydrophobic surface structures on nanoscale surface, which is a promising surface modification method at present [81,82,83,84,85]. The Sol–gel coating can be produced by electrophoretic deposition, impregnation, and sputtering. At present, the deposition coating mainly uses silica sol, which is composed of alkoxy compound and its composite material with metal salt solution. Hydrophobic coatings can be formed directly by introducing a hydrophobic agent (hexamethyl-disilazane, trimethylchlorosilane) into the aerosol and curing it on the material surface. In recent years, the preparation of hydrophobic and super-hydrophobic surfaces has received extensive attention, among which the use of organosilicon fluoro compounds, especially containing hydrolytic groups, is the key development direction [86,87,88]. Fluorinated compounds can be used not only as modification additives in the preparation of Sol–gel composites used for coating formation, but also as the main components of hydrophobic coatings [89].

According to different materials and application scenarios, a large number of surface modification methods have been proposed. Surface modification is a popular research field, especially super-hydrophobic surfaces (which provide a wet angle of more than 150 degrees), including Nano-surface [86,87,90], which has been a new research hotspot in the past two decades [74,89,91]. The surface modification method of PDMS mentioned above is also applicable to common microfluidic materials such as plexiglass. For example, hydrophobic glass can be obtained by preparing the surface of glass by Sol–gel method [92]. There are also other methods, such as ultraviolet (UV) treatment [93,94], chemical vapor deposition (CVD) [95,96], self-assembled monolayers (SAMs) coatings, etc.

By changing the contact angle of the material through any of these methods, the Laplace pressure of the liquid interface is changed. This shift in Laplace pressure is essential for the manufacture and design of the CPCV.

#### 2.2.4. Partial Hydrophilic/Hydrophobic Treatment

**Figure 2:**
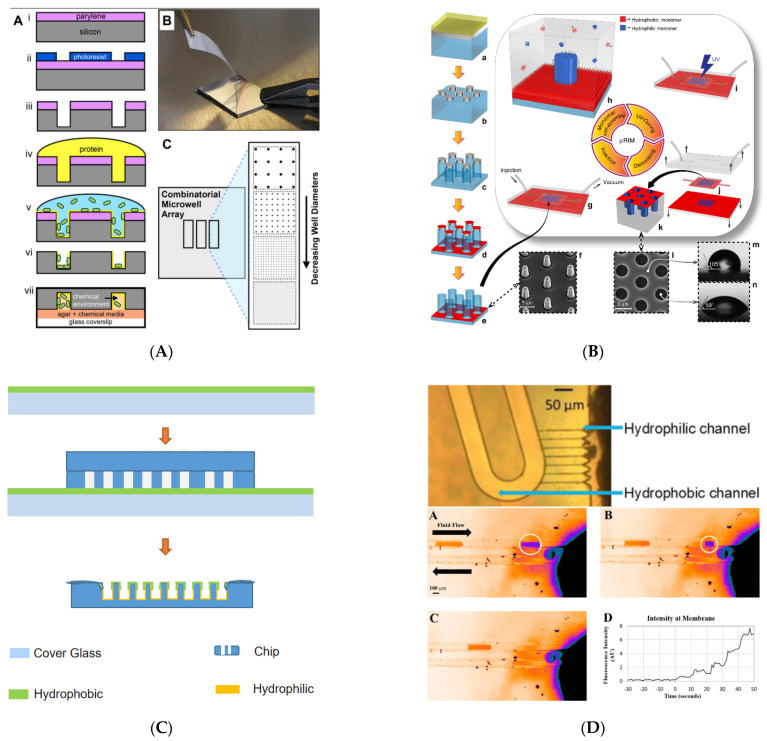
Some examples of surface treatment. (**A**) Microwell arrays for bacteria stochastic assembly. Reprinted with permission from [97]. (**B**) Hydrophilic in-hydrophobic femtolitre-well arrays. Blue color indicates hydrophilic surfaces; red color hydrophobic surfaces. Reprinted with permission from [98]. (**C**) Spin coating liquid hydrophobic liquid to make surface and part of chip wall hydrophobic. (**D**) Local hydrophilic/hydrophobic treatment silicon chip used in sampling (the top half part) and hydrophilic membrane used in microfluidic droplet extraction (the lower half part). Reprinted with permission from [80,99].

By coating the parylene layer and etching it with a designed pattern, the chip surface can be hydrophobic. While the bottom of the microcavity is still hydrophilic, the parylene layer also can be peeled off to restore surface hydrophilicity (Figure 2A). In another article, silica pillars were coated with Cr and Teflon on top (red). During the lift off process, only the top of silica pillars recovered hydrophilicity because Cr was only coated on the top of pillars (Figure 2B). Local hydrophilic/hydrophobic property also can be obtained by transferring the coating from plane to rugged structure (Figure 2C). To extract aqueous droplets from oil, some local hydrophilic/hydrophobic structures were built. In one work, Sigmacote (Sigma Aldrich, Burlington, MA, USA) was used to render the channels hydrophobic upon further baking at 120 °C for 1 h. After that, the capillaries were brought back to a hydrophilic state by stripping the silane layer. This is done by flowing 2-propanol at both the inlet (10 kPa) and the outlet (−100 kPa) through the 50 μm channels while drawing 0.1 M potassium hydroxide through the capillaries. In a similar work, a piece of membrane was bonded with a droplets generation chip made of cyclic olefin copolymer (COC) to extract aqueous droplets from oil (Figure 2D).

### 2.3. Channel Shape

#### 2.3.1. Straight Microchannel

The principal radii of curvature R from Equation (1) may be different depending on the shape of the microchannel. If the microchannel is a closed cylinder with radius *r*, the fluid boundary meniscus in the channel is relatively static, and the two principal radii of curvature of the meniscus are equal, substituting $R=r/cos\theta_{c}$ into Equation (1), the Laplace pressure of the cylindrical channel can be obtained as follows:

(4) $$\Delta P=\frac{2\gamma }{r}cos\theta_{c}$$

where $\theta_{c}$ is the contact angle for the fluids at the solid boundary. Due to the extensive use of simple molding technology, a large number of microfluidic devices are rectangular channels. The equation can be modified to a 3D model as [50]:

(5) $$\Delta P=2\gamma (\frac{1}{w}+\frac{1}{h})cos\theta_{c}$$

where, the curvature radius of the height *h* direction in the microchannel is $R_{h}=h/\left(2cos\theta_{c}\right)$, and the width w direction is $R_{w}=w/\left(2cos\theta_{c}\right)$.

#### 2.3.2. Shape Change Microchannel

In a closed hydrophilic microchannel, by gradually increasing the cross section of the channel, the meniscus in front of the liquid will be eliminated and the additional Laplace pressure generated by the contact angle can be offset. The details of several gradually widening walls are summarized below.

In the rectangular channel, the gradual expansion of the channel can be carried out along the two directions of width and height. In microchannel, the triangular expansion angle $\beta_{0}$ without attachment pressure should meet the following requirements:

(6) $$\Delta P=\frac{2\gamma }{h}\left(\frac{cos\theta_{c}-\frac{\alpha }{sin\alpha }sin\beta }{cos\beta +\frac{sin\beta }{sin\alpha }\left[\frac{\alpha }{sin\alpha }-cos\alpha \right]}\right)\Delta P=2\gamma (\frac{1}{w}+\frac{1}{h})cos\theta_{c}$$

The angle of triangular expansion is β. When β < $\beta_{0}$, the liquid level meniscus is concave, and liquid advances, wetting the surface. When β > $\beta_{0}$, the liquid level meniscus protrudes, and Laplace pressure creates a pressure barrier, stopping the flow of liquid (Figure 1B).

If the microchannel height *h* is much bigger than the width w, then the meniscus can be regarded as one-dimensional. The meniscus contour is considered as a section of sphere with an arc angle of 2α. Man et al. give the pressure barrier *P* caused by 2D geometric change in the case of channel mutation [47]:

(7) $$\Delta P=\frac{2\gamma }{h}\left(\frac{cos\theta_{c}-\frac{\alpha }{sin\alpha }sin\beta }{cos\beta +\frac{sin\beta }{sin\alpha }\left[\frac{\alpha }{sin\alpha }-cos\alpha \right]}\right)$$

The expression derived by Chen et al. gives the pressure model of the geometric expansion of the micro-channel in the 3D model [100]:

(8) $$\Delta P=\frac{2\gamma }{w}\left[\left(-\frac{w}{h}\right)cos\theta_{c}+\left(\frac{cos\theta_{c}-\frac{\alpha_{w}sin\beta }{sin\alpha_{w}}}{-cos\beta +\frac{sin\beta }{sin\alpha_{w}}\left(\frac{\alpha_{w}}{sin\alpha_{w}}-cos\alpha_{w}\right)}\right)\right]$$

Figure 1C is a top view of the model. *β* is the angle of expansion in the width *w* direction. The depth *h* remains constant in the whole channel. The $\alpha_{w}$ is circular arcs angle in width directions.

Jerry M et al. undertook an extensive analysis of the axisymmetric channel pressure barrier with sudden expansion (by suddenly increasing the cross section of the channel), along the channel direction and gave the expression [56]:

(9) $$\Delta P=\frac{-2\gamma }{R+x_{1}tan\beta }\left[\frac{cos\theta_{c}-\frac{2sin\beta }{1+cos\alpha }}{cos\beta -\frac{sin\beta }{{sin}^{3}\alpha }\left(2-3cos\alpha +{cos}^{3}\alpha \right)}\right]$$

As shown in Figure 1D, the axially symmetric channel radius of the column is *R*, and the wetting length is $x_{1}$. The meniscus shape, defined by $\alpha =min(\pi /2-\theta_{c}-\beta ,-\pi )$, is held invariant throughout this regime. When $\beta =90^{{}^{\circ}}$, the meniscus shape is described by $\alpha =-\theta_{c}$. The formula is as follows:

(10) $$\Delta P=\frac{2\gamma sin\alpha }{R+x_{1}}$$

Figure 1E shows the liquid pressure in hydrophilic (Figure 1E(i)) and hydrophobic (Figure 1E(ii)) microchannels with a radius of 300 μm as a function of liquid volume for expansion angles in the range β=0–90°. For a straight channel (β=0), the pressure in hydrophilic microchannels is maintained at a positive constant of 195 Pa driving the liquid downstream without being blocked, the pressure in hydrophobic microchannels is negative (−195 Pa) indicating that an external force is required to propel the liquid forward. As the meniscus arrives at the edge where sudden expansion in the cross section begins, the pressure drops rapidly to reach a minimum, followed by a steep increase. The liquid is blocked by the pressure barrier formed at the sudden opening and an increase in liquid volume needs an extra force. In other words, a sudden expansion of the microchannel can act as a valve to control the flow of liquid in the channel. In hydrophilic pipe, if β is small (β < $\beta_{0}$), the falling pressure of the liquid is still positive, and the Laplace pressure continues to push the liquid forward, it is a valveless state. Note that except for the channels with large expansion angles ($\beta +\theta_{c}>\pi$), threshold pressure increases with an increase in expansion angle.

### 2.4. Examples of the CPCV

In microfluidics, a series of valves with unique structure and function are manufactured using the principle above. There are many examples to show the control valve applications [101,102,103,104,105,106,107,108,109,110,111]. According to the different functions of valves, CPCV includes stop valve, trigger valve, and delay valve [112].

#### 2.4.1. Stop Valves

The purpose of the stop valve is to temporarily block the flow of liquid in the capillary. A stop valve can be formed by reasonably changing the structure of the meniscus in the microchannel to generate the additional pressure we need and block the liquid flow. There are two main ways: the first kind of valve depends on changes in the hydrophilic and hydrophobic properties of the microchannel surface, such as the hydrophilic microchannel surface which is coated with hydrophobic materials [45,113,114]. The second type of capillary valve is fairly simple to manufacture and does not require additional surface treatment to alter the microchannel hydrophilicity. The liquid is simply stopped when the microchannel cross section changes abruptly (hydrophilic microchannels expand suddenly or hydrophobic microchannels shrink suddenly) [46]. These stop valves can stop the flow of liquid in microchannels without external forces and have been successfully used in the partially wetted regime.

**Figure 3:**
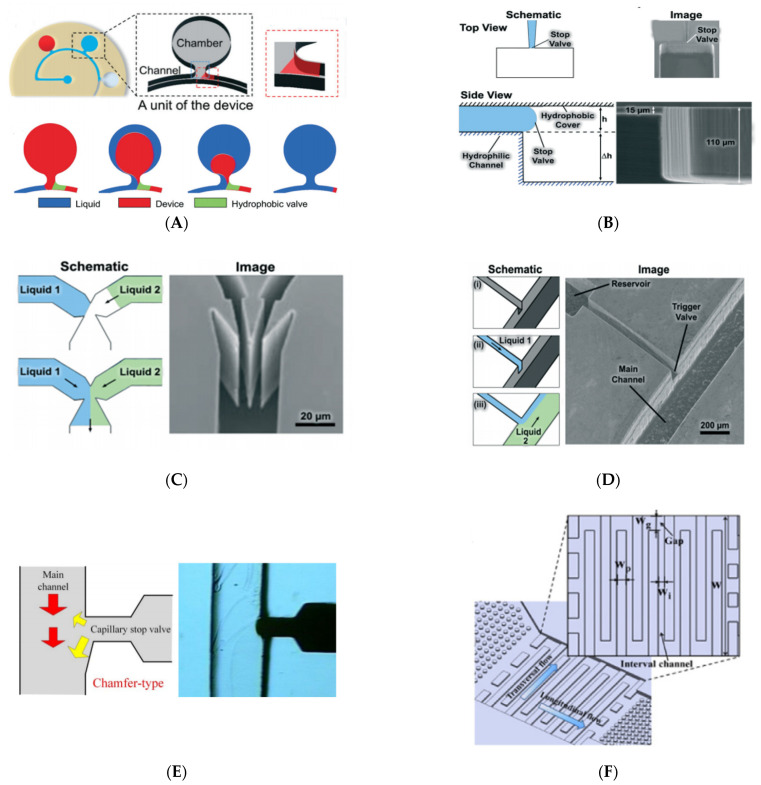
Structures of CPCV. (**A**) Surface treatment type stop valve. Reprinted with permission from [45]. Copyright 2020 Royal Society of Chemistry. (**B**) Cross section expanding type stop valve. (**C**) Single layer trigger valve. (**D**) Stair-step trigger valve. ((**B**–**D**) reprinted with permission from [14]. Copyright 2018 Royal Society of Chemistry.) (**E**) Chamfer-type trigger valve. Reprinted with permission from [15]. (**F**) Comb-like delay valve. Reprinted with permission from [18].

**Surface treatment type stop valve**. Go Takei et al. designed and manufactured a four-step wettability passive surface tension stop valve with a pressure barrier of 6.8–12.5 kPa based on the characteristics of titanium dioxide nanoparticles, and proposed a method for microfluidics to control the surface wettability of microchannels [113]. The super-hydrophobic surface can be obtained by hydrophobic treatment on the rough surface of titanium dioxide. The surface wettability changes from super-hydrophobic to super-hydrophilic by photocatalytic decomposition of hydrophobic molecules under controlled light irradiation. The wettability pattern of the channel surface can be flexibly drawn, exposure can be controlled by graphics, and wettability can be adjusted over a wide range. Yingxue Zhang et al. used a surface tension stop valve that can be used in collecting skin sweat. The valve is positioned at the interface between the single-opening liquid collection chamber and the microfluidic channel to ensure the collection of sweat and reduce the evaporation of sweat [45]. As shown in Figure 3A, when sweat enters the hydrophilic microchannel, the stop valve forms a pressure barrier to prevent sweat from entering the back channel and forcing sweat into the collecting chamber. When the sweat fills the chamber completely along the wall, the stop valve will not work and the liquid passes over the stop valve.

**Cross section expanding type stop valve.** The liquid flow in the microchannel is stopped by a sudden expansion in the channel geometry without external intervention. The normal stop valve stops the liquid only by expanding the width of the microchannel. This single-layer method is very simple and reliable in active hydrophobic systems. A two-level stop valve was designed and manufactured by Gliere and Delattre using hydrophilic microchannel and hydrophobic PDMS covers, and the threshold pressure and microchannel size were theoretically analyzed and experimentally verified (Figure 3B). A two-level stop valve with channel cross section extending along the width and depth was prepared by reactive ion etching technique. Tests on the 15 µm × 15 µm valve confirmed that the valve can block the buffer normally and it opens at a burst pressure within the range of 1–10 kPa [46].

#### 2.4.2. Trigger Valves

Stop valves can be easily converted into trigger valves by cross-setting two microchannels. The flow of the first microchannel stops flowing at the intersection of channels, and the stopped liquid flow can be restored when the second liquid reaches the intersection.

The trigger valve can be assembled by stop valves to allow the stopped liquid to resume flow due to the subsequent trigger liquid [115,116]. For microfluidic applications requiring accurate control of reaction time and liquid flow, ensuring that multiple reagents reach the reaction chamber simultaneously is the core of the problem. If the reagents that should have reached the cross junction of the channel do not arrive at the same time, bubbles are often generated between multiple reagents because the inlet and outlet channels are directly connected to the reaction chamber. These bubbles may change the planned flow path of the reagent or directly truncate the channel [52]. Considering the principle of the stop valve at the junction of multiple channels, the trigger valve is designed to ensure that a single fluid stops at the valve, and the flow will be resumed only after the arrival of the second liquid, which can effectively avoid the bubble problem existing in the microchannel.

The geometry of the trigger valve determines its function. The basic structure consists of two inlets and one outlet in a shape similar to the Y-junction (Figure 3C). When the liquid from the two inlet reaches the connection point alone, the Laplace pressure of the liquid increases due to the abrupt change of channel cross section, and the Y-junction acts as a geometric stop valve. However, when more than one inlet liquid is present at the junction, the valve allows the liquid to pass through the exit, because liquid contact rebuilds the original liquid meniscus, and the Laplace pressure of the newly formed meniscus is not enough to stop the liquid. These trigger valves can hold liquid in an inlet for an average of 15 min, but require a very high aspect ratio to successfully stop the liquid [14].

Yaw-Jen Chang et al. introduced the chamfered side structure into the trigger valve [15], as shown in Figure 3E. As a flow control device, there will be a countercurrent phenomenon in the commonly used T-type trigger valve, and the use of chamfer can alleviate this phenomenon. The chamfered side of the valve not only holds the reagent in place, but also increases the contact area between the reagents. Due to chamfering, the stop reagent meniscus is more likely to be triggered by reconstruction, and the reagent is also more likely to be pulled into the main channel.

The stair-step triggered valve with lower aspect ratios designed and discussed by Zhang Lei et al. is shown in Figure 3D. Instead of two microchannels narrowing into a point, the stair-step trigger valve microchannels meet at an orthogonal intersection. The stair-trigger valve is sufficient to stop the liquid for more than 30 min and is more reliable than the single level trigger valve. However, a single level trigger valve can stop either liquid until both are present while the stair-trigger valve can only stop one liquid, which seems more useful. The valve channel, with sudden expansion along the width and depth of the channel, is fabricated on silicon using a two-step etching process. The two entrances of the stair-step trigger valve are non-equivalent. The function of the stair-step trigger valve is to stop the flow of the liquid from the cut-off passage and allow the liquid from the triggered passage to pass through the stepped structure normally [19]. Chen, Xi et al. used polyethylene glycol (PEG) and other materials to modify the surface to form the stair-step liquid trigger valve, and further evaluated and discussed the reliability of the valve [117].

#### 2.4.3. Delay Valves

For some microfluidic systems, it is necessary to accurately control the arrival time of reagents. This can be accomplished with delay valves.

The delay valve controls the liquid flow time by increasing the flow resistance which decreases the liquid velocity or increasing the channel length to increase the wetting length [18,42]. In order to reduce the velocity of the liquid, it is possible to add hydrophobic marks in the channel to increase the resistance.

The moving speed of the filling liquid front in the wide microchannel is lower than that in the narrow microchannel. M. Zimmermann et al. achieved two parallel flow paths by simply combining the smaller channel with the larger one. At the same time, they used the guide structure to prefabricate the sequence of liquid entering each area, so as to achieve the purpose of delay [112].

Ji Won Suk et al. proposed a simple method for making the delay valve using an array of hydrophobic patterns to control the liquid velocity to reach the purpose of delay. The whole microchannel is composed of a hydrophilic floor and a hydrophobic roof. In order to reduce the liquid flow rate, hydrophobic patches are created on the floor. By adjusting the location, number, and spacing of hydrophobic patches, the flow rate of liquid throughout the channel network can be customized [42].

The comb-like delay valve proposed by Jingmin Li et al. (Figure 3F) provides a delay range from tens of seconds to several minutes, providing accurate time control for the reaction between samples and reagents. The delay time of the delay valve can be changed by adjusting the number and layout of the comb protrusions. As the simple structure of the valve and the minimum line width of the comb protrusions is over 100 μm, it can be easily manufactured in stainless steel molds and used in mass production [18]. The capillary stop valve, capillary retention valve, also belong to this part.

## 3. Applications and Impacts of CPCV

With the diversification of microfluidic chip application scenarios, the function of microfluidic chip is more and more powerful, and the role of valves is more and more important. As one of the most unique valves to control the flow of microfluidic equipment, the CPCV is valued for its simple design and customizable nature. CPCV can be easily integrated into different applications by designing microchannel structures or modifying hydrophilicity and will shine with the development of microfluidic technology. A brief overview of valve applications in different scenarios is listed as below, which includes but is not limited to making easy automation of microfluidic networks; making broader use of microfluidics by open channel; sampling and delivery in a controlled manner.

### 3.1. Simplifying Automation of Microfluidic Networks

The use of CPCV greatly expands the application of microfluidic networks and increases the automation of microfluidic chips [14]. It has accelerated the automation of microfluidic chips combined with the point of care testing (POCT) instrument for the automated detection of disease, which is easily broadly applied and commercialized.

#### 3.1.1. Microfluidic Networks

A simple microfluidic network was constructed by Olanrewaju, A. O et al., in order to achieve rapid and simple bacterial detection. The trigger valve is used to isolate the preloaded sample and the two test reagents, and the buffer solution is added to trigger the automatic analysis of the bacterial test without human intervention [118].

**Figure 4:**
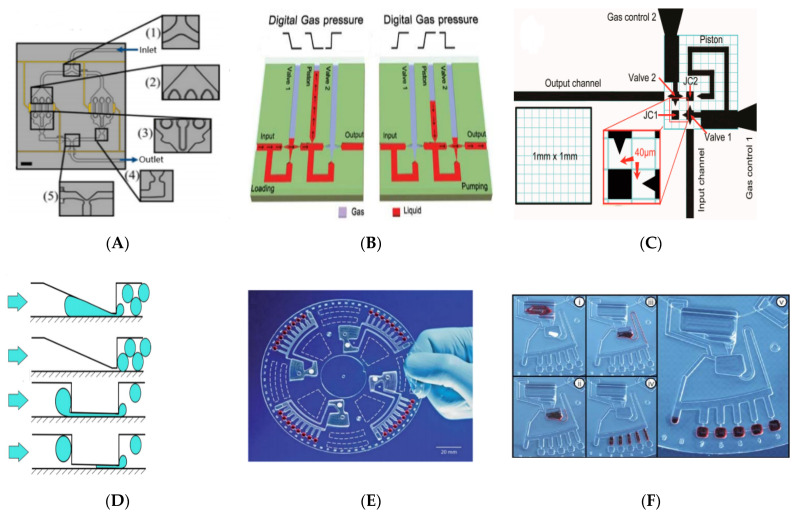
Application of CPCV in different microfluidic scenarios. (**A**) CPCV is used to realize the bubble-free microfluidic network for filling. Reprinted with permission from [119]. Copyright 2014 American Institute of Physics. (**B**) Micropump using CPCV components. Reprinted with permission from [120]. Copyright 2011 Royal Society of Chemistry. (**C**) The optimized micro pump with an area of one square millimeter. Reprinted with permission from [121]. (**D**) Structure of droplet generator using CPCV principle. Reprinted with permission from [122]. Copyright 2017 Royal Society of Chemistry. (**E**) A panoramic view of the lab on disk chip. Reprinted with permission from [123]. Copyright 2010 Royal Society of Chemistry. (**F**) The process of working in a lab on a disk chip. Reprinted with permission from [124]. Copyright 2010 Royal Society of Chemistry.

With the expansion of microfluidic networks, bubble-free filling is a key prerequisite. By using CPCV to optimize the entire microfluidics network and pre-determine the flow of liquid through the device, bubbles within the microfluidic structure can be effectively avoided. As shown in the Figure 4A, CPCV can be set at each node of the microfluidic network, such as the merging or splitting of channels and the inlet and outlet of the assembly chamber. By setting valves at the nodes, the flow of the nodes in the network can be controlled and the reagents filling order for the entire network can be customized. With the cascade of valves, the driving pressure of the whole network increases continuously, and it needs to be established through several stages to realize the bubble-free filling of the hydrophobic microfluidic device [119].

#### 3.1.2. Micropump

Professor Peng applied the principle of CPCV to assemble a micro surface tension pump [120], the pump can pump liquid at a speed of 10 nL·s^−1^ into a 300 µm wide microchannel by pneumatic control. As shown in Figure 4B, during the pump loading process, valve 1 (inlet valve) is opened at low pressure and valve 2 (outlet valve) is closed at high pressure, as Laplace pressure fluid passes through valve 1 into the intermediate piston section. During pumping, valve 1 is closed and valve 2 is opened, using high pressure to pump the liquid from the piston through valve 2. In 2016, Professor Peng optimized the above pump (Figure 4C). By combining the pneumatic control of the input valve and the piston into one channel and simplifying the channel, the original three-way control was reduced to a two-way control and the compressed pump area was reduced to 1 square millimeter, which greatly enhanced the fault-tolerance and recoverability of the pump [121].

#### 3.1.3. Droplet Generation

Accurate generation of micro droplets is a hot topic for researchers in the microfluidic laboratory. Zhu, Pingan and Wang, Liqiu reviewed in detail the various active and passive methods now used for droplet generation [125]. The T-shaped structure is the most common droplet generation method. When mutually insoluble fluids meet at the intersection of vertical T-shaped channel, under the action of pressure and shear force, the continuous phase truncates the dispersed phase, which can simply disperse the dispersed phase droplets in the continuous oil phase. In addition, CPCV-like structures are widely used to generate micro droplets [122,126,127,128]. For example, the structure (Figure 4D) used by Postek, W. et al., through a gradually narrowed microchannel or slit, can further disperse larger droplets to produce nanoscale droplets whose size is largely determined by the geometry of the device and which do not require additional external phase flows [122].

#### 3.1.4. One Example: Lab on a Disk

In the last 10 years, the field of centrifugal microfluidics has experienced tremendous development, and its powerful and high-performance liquid handling capabilities have led to the widespread use of modular, multipurpose lab-on-a-disc platforms in the life sciences [41,48,129,130]. CPCV is often used to separate the measuring chamber from the reaction chamber in centrifugal microfluidics [123,131], such as the work by Lutz, Sascha et al. [124]. As shown in the Figure 4E,F, after adding the specimen and sealing the inlet hole, the disc will rotate at a frequency of 6.6 Hz, the sample will be calibrated in five adjacent metering chambers, and the excess liquid will be collected in an adjacent waste reservoir located behind the metering chambers, during which the CPCV prevents the sample from entering the reaction well. When unidirectional and alternating rotation frequencies between 6.6 and 27 Hz are applied, the CPCV is opened, and the sample enters the reaction well until it is filled. Then, the system enters a stable equilibrium state, and the rotation frequency will not affect the position of the equable sample. CPCV with similar structure is widely used in clinical chemistry, immunodiagnostics and protein analysis, cell processing [129], medical diagnosis [132], and other fields.

### 3.2. Various Valve Actuation Mode

As a kind of passive valve, CPCV can stop the liquid in the valve without external force. That is, the valve is in the closed state. However, opening the valve to allow the fluid to pass normally requires additional force to break the meniscus in order to completely wet the channel. This additional force can be introduced in a number of ways, the most commonly mentioned being the capillary burst valve [36]. There are many microfluidic devices that use external pressure directly to open the valve [114]. This approach is simple and straightforward but requires additional equipment such as injection pumps and does not reflect the driving advantages of the CPCV. The following are brief introductions of some other driving common modes of CPCV.

#### 3.2.1. External Forces

Using Laplace pressure to directly drive liquid (such as water in the hydrophilic channel, oily liquid in the hydrophobic channel) to rebuild and reconstruct the meniscus to open the valve is the most ideal method to open CPCV. This completely passive way does not require external forces or any external equipment, and only depends on the channel structure and the liquid properties, which is easy to implement and extremely conducive to the miniaturization of equipment. Due to the deterministic structure and passive triggering, these trigger valves lack flexibility in opening. When the valve is fully open, it needs to drain all the fluid in the channel and then close the valve to restore it to its original state, which lacks reusability. However, its disposable features, due to its low cost, are more promising in real-time diagnosis, biological analysis, and other aspects.

**Figure 5:**
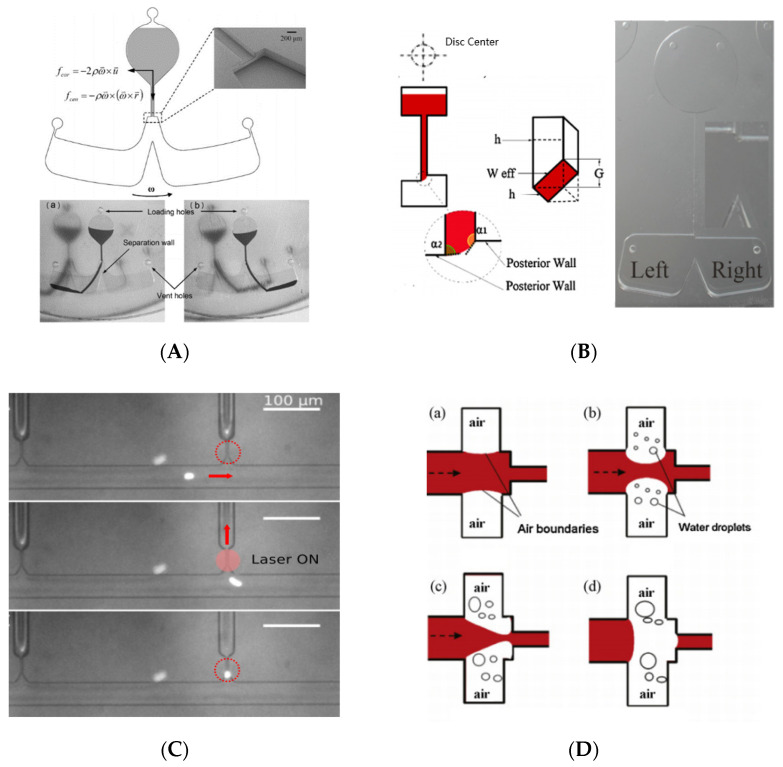
Different modes of valve actuation. (**A**) Centrifugal force drives CPCV by changing the direction of rotation to allow the liquid to enter different chambers. Reprinted with permission from [133]. (**B**) Asymmetric structure, centrifugal force driven, changing the speed of liquid into different chambers. Reprinted with permission from [134]. (**C**) The laser drive is used to capture particles. Reprinted with permission from [135]. Copyright 2017 American Institute of Physics. (**D**) An example using thermally driven CPCV. Reprinted with permission from [136].

Centrifugal force can be used not only to open the CPCV, but also to control the flow direction of the centrifugal microfluid after opening the valve. For symmetrical structures, as shown in Figure 5A, the liquid is driven into the selected chamber by changing the direction of rotation [133]. Amin Kazemzadeh et al. designed an asymmetric structure (Figure 5B), which can control the flow direction of centrifugal fluid without changing the rotation direction and without using external sources or surface treatment. The device is related to the speed or frequency of rotation. At low speeds, because of the asymmetric structure of the valve, the liquid is directed in one direction along the microchannel. At high speeds, the liquid is directed in the opposite direction by Coriolis forces [134].

We know that the surface tension forming the meniscus creates a pressure barrier that can stop the capillary flow. As a valve, it is a good idea to use the centrifugal force generated by spinning to break through the pressure barrier. For example, Lab-on-CD devices make the chip rotate through a simple platform. They use the Laplace barrier generated by surface tension to prevent the liquid flow in the microchannel, and use the centrifugal force generated by rotation to drive the liquid to break through the pressure barrier and control the liquid flow [137,138]. CPCV will generate different threshold pressure barriers with different channel sizes and shapes. When CPCV structure is fixed, the threshold speed of the Lab-on-CD device can be calculated by CPCV position, reagent density, and reagent column length in the microchannel. When the rotating speed of the platform exceeds the threshold value, the centrifugal force generated is greater than the barrier pressure, the meniscus burst liquid enters the expansion space, and the valve is opened. According to the change of interface energy, Jerry M et al. give a simple formula to calculate the critical rotational speed and burst pressure to overcome the CPCV threshold pressure at the sudden expansion of the rectangular channel section [50]. By setting the CPCV of different thresholds and accurately controlling the rotation speed, it is possible to integrate a variety of functions into Lab-on-CD devices, such as flow sequencing, cascade micro-mixing, capillary metering, and so on [139,140]. For example, the sample separator designed by Leu, T. S. et al. using density gradient centrifugation to segment the samples liquid in the microstructure. The different density segments required by the sample can be simply separated by rotating at different speeds [141].

#### 3.2.2. By Changing Surface Tension

Londe, G et al. made thermos-sensitive CPCV using a switchable thermos-sensitive polymer [142]. At room temperature, the surface of thermos-sensitive polymer is hydrophilic and allows water to flow. When the temperature exceeds 65 degrees Celsius, the surface becomes hydrophobic, thus inhibiting water flow. Recently, L. Li et al. grafted thermo-responsive polymer Poly(Nisopropylacrylamide) (PNIPAm) onto PDMS. When the channel temperature increased from 20 to 37 °C, the channel surface changes from hydrophilic to hydrophobic, forming a CPCV [143]. Thus, the temperature control valve can regulate sample flow more flexibly. Due to the interrelationship between fluid surface tension and temperature, increasing the temperature can make the CPCV meniscus rupture. Johan Eriksen et al. embedded the near-infrared absorption dye film into a sealed microfluidic device and used laser local heating to open a single valve [135], shown in Figure 5C.

Wei Xu et al. proposed a basic valve design using trapped air to control flow. The small concave structure was made in the micro channel at the same position as the CPCV section expansion, as shown in Figure 5D. When the liquid filled the micro channel, due to the hydrophobicity of the manufacturing material PDMS, the surface tension limited the fluid into the concave chamber, and the hydrophobic chamber intercepted the air to form air bubbles. By increasing the chip temperature, the bubbles gradually expanded and moved into the main channel, limiting the flow of fluid in the channel. Finally, the bubble completely truncated the main channel and blocked the flow of liquid [136].

### 3.3. Make Broader Use of Microfluidics by Open Channel

Open channel microfluidics devices usually lack at least one limiting liquid physical sidewall or cap. By applying the hydrophilic and hydrophobic properties of the channel and the surface tension of the liquid, the liquid is fixed to flow along a specific path [144,145]. The open channel microfluidic device is a special application of CPCV using the theory from Section 2.4.1. Compared with traditional closed microfluidic structure, the open channel microfluidic structure has obvious advantages and disadvantages. As the top is open or semi-open, it facilitates the entry of sample reagents and eliminates the air-bubble problem in traditional closed wall microsystems. At the same time, since there is no cover at the top, it is easier to manufacture. However, the top opening also greatly increases sample evaporation and the risk of reagent contamination. In addition, the open structure greatly restricts the use of the drive system [146,147].

**Figure 6:**
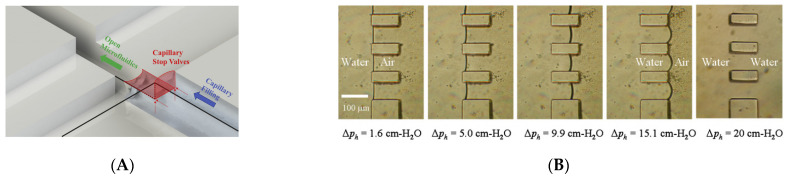
Application of CPCV in different microfluidic scenarios. (**A**) Use of CPCV to quick rivet antibody with open channel. Reprinted with permission from [148]. (**B**) Use of CPCV to build virtual channels. Reprinted with permission from [149]. Copyright 2011 American Institute of Physics.

V.A. Papadimitriou et al. used CPCV to realize the fast and convenient immobilization of antibodies after chip bonding. This method can automatically and conveniently shape the liquid in a closed chip. The basic structure is shown in Figure 6A, a deep channel is placed across another shallow channel, the deep channel is filled with reagents containing antibodies, and the shallow channel perfusion is used to detect the samples. The reagent fills the deep channel rapidly due to capillary force, and when it reaches the intersection of the deep and shallow channels, the upper part of the reagent in the deep channel stops flowing because of the sudden opening of the channel, which is bound by Laplace pressure. The lower part of the reagent is free to flow through the trench below the shallow channel. When the reagent passes through the trench through the shallow channel, the capillary force pulls the reagent to fill the whole deep channel. Throughout the process, the reagents are fixed to the bottom and vertical sides of the intersection of the deep and shallow channels, which functions like a globe valve [148].

Hsuan-hong Lai et al. used the characteristics of CPCV to design the virtual walls with a range of feature sizes [149]. As shown in Figure 6B, the virtual wall is composed of a row of rectangular PDMS micro-columns, and the two rows of parallel virtual walls constitute the channel, and then the device is divided into an intermediate liquid channel and an air chamber on both sides. When the liquid is filled, because of the Laplace pressure of the liquid, the liquid is confined within the middle channel, and the air is squeezed into chambers on both sides of the channel. When the hydraulic pressure is enhanced, the liquid between the micro-columns will advance towards the air chamber on both sides; when the pressure decreases, the liquid will contract towards the middle channel; when the pressure exceeds the threshold, the liquid passes through the virtual wall composed of PDMS micro-columns and completely fills the air chamber. To restore the virtual wall, the device needs to be cleared of all fluids and started at low pressure.

### 3.4. Sampling and Delivery with Controlled Manner

Sample collection and delivery are two indispensable functions of microfluidic chips. The chip contact tip must first enter into the sample and then sample or deliver and transport along a given route in a controlled manner, which is the basic guarantee for the implementation of a microfluidic probe. CPCV can help to achieve these functions based on the theory from theory 3.1.1. and sometimes with theory 3.1.3. It can transport and stop the flow as well as protect the sampling or delivery microenvironment at the chip contact tip by forming the safe barrier of hydrophilic features where only water phase can go through rather than oil/gas phases in a controlled manner.

**Figure 7:**
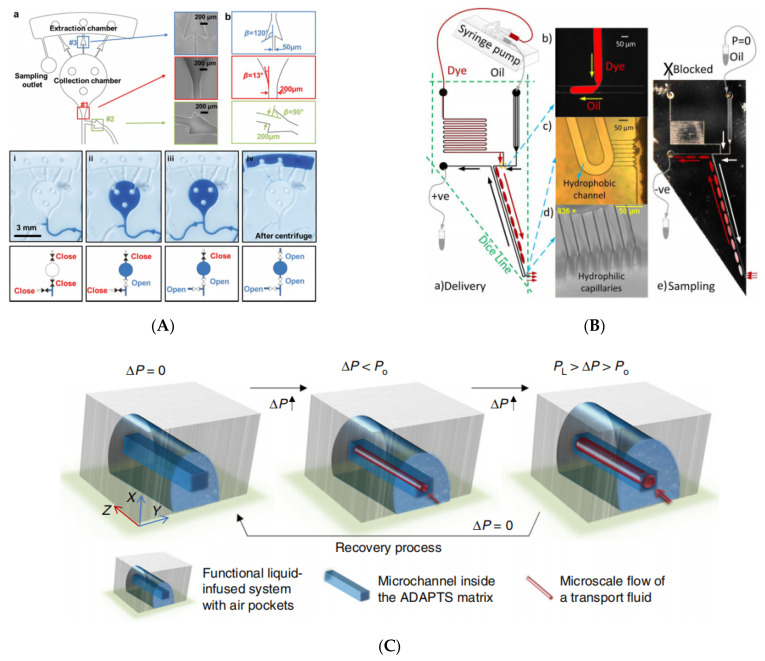
Use of CPCV in sampling and delivery. (**A**) A sweat sampling microfluidic device that adheres to the skin. Reprinted with permission from [150]. Copyright 2017 Wiley-VCH. (**B**) A needle-shaped liquid sampling and delivery device. Reprinted with permission from [80]. Copyright 2017 American Institute of Physics. (**C**) Surface tension applied in an adaptive air/liquid pocket transport system (ADAPTS) where the microscale flow (shown in red) is in the square microchannel (shown in dark blue). Reprinted with permission from [151].

Choi, Jungil et al. designed a skin microfluidics device with accurate sampling capability, as shown in Figure 7A, using a set of carefully designed CPCVs to guide sweat to fill the micro reservoir in a sequential flow [150]. The initial liquid arriving at CPCV1 and CPCV2 meets them in a closed state. When the breakthrough pressure of CPCV1 is reached or exceeded, CPCV1 opens, allowing fluid to enter the chamber. After this chamber is filled completely, CPCV2 is broken down under sufficient pressure. The breakthrough pressure of CPCV2 is lower than that of CPCV3. Through this process, all chambers are sequentially filled with fluid at pressures greater than the CPCV2 breakthrough pressure. Then, the chip is removed from the skin and placed into the centrifuge. CPCV3 is turned on by centrifugal force and the sweat is moved to the reaction chamber behind the micro reservoir for experimental analysis. This design of CPCV satisfies that the pressure generated by sweat glands exceeds the breakthrough pressure of CPCV1 and CPCV2 that ensures sweat can enter and fully fill the micro reservoir. As the pressure generated by centrifugation exceeds the breakthrough pressure of CPCV3, sweat can enter the reaction chamber.

Based on the principle of CPCV, a needle-shaped microfluidic device is proposed, as shown in Figure 7B, which can provide the sampling and delivery of nanoliter solution in droplets [80], where CPCV serves as the safe barrier for a safe sampling and delivery microenvironment. The device consists of a T-junction forming droplets. The needle was formed by a parallel hydrophobic channel, and the sampling tip was formed by seven hydrophilic microchannels (similar to CPCV). During the delivery process, droplets dispersed in the oil phase produced by the T-junction move along the channel under pressure at the needle tip, where the tip (seven capillaries) is hydrophilic and the droplets can pass through with a pressure gradient close to zero but the mineral oil requires 19 kPa pressure. During the sampling process, a negative pressure is added to the device to allow the liquid to enter the device from the tip, create droplets in the oil phase and move with the pressure gradient. This needle was applied for H_2_O_2_ detection [152]. The similar principles have been widely applied by Feng on the membrane on the plastic chip [99]. It has been further reviewed by this type of principle used in the sampling probe [153].

Xu, Hou et al. presented an adaptive air/liquid pocket transport system (ADAPTS) [151] with solid porous structures and liquid/ gas interfaces, which are suitable for low-pressure applications. Initially, functional liquid is filled in the porous structures. When the transport fluid enters, it displaces the functional liquid to open a flow path in the microchannel (Figure 7C). By then, the applied pressure (ΔP) is bigger than the threshold pressure (Po). The functional liquid can refill the microchannel after removal of the pressure and recover to the original state immediately.

## 4. Conclusions and Outlook

In this paper, the principle, composition, and application of CPCV were briefly reviewed. In microfluidics, CPCV is used as flow controller and forms important parts of microfluidic chips. CPCVs are generated by additional pressure due to meniscus variations in microchannels. The characteristics produced by it reflect its advantages and disadvantages. The CPCV does not need additional equipment; the switching pressure is convenient, controllable, and only related to its structure; it is simple to manufacture, convenient to integrate and put on scale production; it has a good prospects in all branches of micro-flow control (biomedical testing, wearable devices, etc.). The disadvantages are also relatively obvious, the valve is more passive and there is a lack of flexibility. If you want to close the valve after opening the valve, you need to drain all the liquid in the channel and restore it to dry, which lacks reusability. In Section 3, Xu Hou has put effort into this, while it can only be used for low pressure applications, but it is still an improvement. The shortcoming is also the direction of further research in the future, which needs to be jointly promoted by the industry.

We hope the CPCV can make automation easy and merge into the point of care instrument, which can help with the commercialization of microfluidics chips, precise medicine and POCT detections, especially in this COVID-19 virus outbreak period. CPCV can facilitate broader use of open channels in microfluidics by eliminating the air-bubble problem in traditional closed wall microsystems. CPCV can be widely used in the bionic microfluidics; CPCV can be widely used in the sampling probes to avoid the gas/oil pollution to the sampling/delivery microenvironment.

In this review, we focused on fundamental physics theories in detail, categorized them based on the theory elements with new applications of each kind, and examined the potential of real applications in industrial and academic fields. We analyzed and categorized the examples from three fundamental elements: surface tension, contact angle, and different microchannel shape. We hope this review can give a direction for using the CACV in various academic and industrial research, which can provide broader applications.
